# Does citalopram effectively treat post-stroke depression? A protocol for systematic review and meta analysis

**DOI:** 10.1097/MD.0000000000020608

**Published:** 2020-06-26

**Authors:** Jing Hu, Li Ma, Zeng-ye Yang

**Affiliations:** a Department of Neurology, Ankang People's Hospital, Ankang; b Department of Neurology, Yanan University Affiliated Hospital; c Department of Neurology, Yan’an People's Hospital, Yan’an, China.

**Keywords:** citalopram, efficacy, post-stroke depression, safety

## Abstract

**Background:**

This study aims to assess the efficacy and safety of citalopram for the treatment of patients with post-stroke depression (PSD).

**Methods:**

We will comprehensively search Cochrane Library, PUBEMD, EMBASE, WorldSciNet, Web of Science, VIP Database, CBM database, and China National Knowledge Infrastructure. The search period is limited from the construction of each database to the February 1, 2020. No language and publication status are limited. Two investigators will independently carry out study choosing, data extraction, study methodological quality assessment, and quality of evidence. A third investigator will help to resolve any disagreements between 2 investigators. RevMan 5.3 software will be employed for statistical analysis.

**Results:**

This study will summarize the up-to-date evidence and synthesize the data to explore the efficacy and safety of citalopram for patients with PSD.

**Conclusions:**

The results of this study may present helpful evidence to determine whether citalopram is an effective management for patients with PSD.

**PROSPERO registration number:**

PROSPERO CRD42020171015.

## 1 Introduction

Post-stroke depression (PSD) is considered as the most frequent and important neuropsychiatric complication experienced following stroke.^[1–5]^ It often affects functional recovery, quality of life, and rehabilitation, and is associated with a variety of disability, morbidity and mortality.^[6–11]^ It is estimated that its incidence ranges from 23.0% to 76.1% in China.^[12]^ Other studies reported that about 20% to 50% patients with stroke suffer from PSD.^[13–14]^ Thus, it is very essential to treat PSD timely and effectively.

Despite a variety of clinical trials have reported that citalopram is effective and safety management for patients with PSD,^[15–34]^ there are inconsistent conclusions at literature levels. In addition, the present level of evidence-based medical evidence is still insufficient because of the limited number of high quality trials and sample sizes. Thus, we hope this study will provide high quality evidence to appraise the efficacy and safety of citalopram for the treatment of PSD.

## 2 Methods

### 2.1 Study registration

This study has been funded and registered through PROSPERO (CRD42020171015). It has been reported base on the Preferred Reporting Items for Systematic Review and Meta-Analysis Protocols statement.^[35]^

### 2.2 Criteria for including studies

#### 2.2.1 Types of studies

All randomized controlled trials (RCTs) that focusing on the citalopram for the treatment of patients with PSD will be included without language and publication time restrictions.

#### 2.2.2 Types of interventions

We will adopt citalopram treatment alone on patients with PSD as experimental interventions.

As for control intervention, it can be any therapies, such as placebo, acupuncture, and any other management. However, we will not consider any treatments including any forms of citalopram.

#### 2.2.3 Types of participants

We will include any participants who had a confirmed clinical diagnosis of PSD. We will not place any restrictions upon the ethnicity, sex, age, and economic status.

#### 2.2.4 Types of outcome measurements

The primary outcome indicator is depression. It can be assessed by any validated scales, such as Hamilton Depression Scale.

The secondary outcomes are anxiety (as measured by any validated tools, such as Hamilton Depression Scale), health-related quality of life (as identified any related indexes, such as 36-Item Short Form Survey), and any expected or unexpected adverse events.

### 2.3 Search strategy

#### 2.3.1 Electronic databases searches

The following electronic databases will be searched comprehensively from the construction of each database to the February 1, 2020: Cochrane Library, PUBEMD, EMBASE, WorldSciNet, Web of Science, VIP Database, CBM database, and China National Knowledge Infrastructure. There are not restrictions on the language and publication time. The complete Cochrane Library search strategy is created and summarized (Table 1). Similar search strategies of other electronic databases will be modified and built.

**Table 1:**
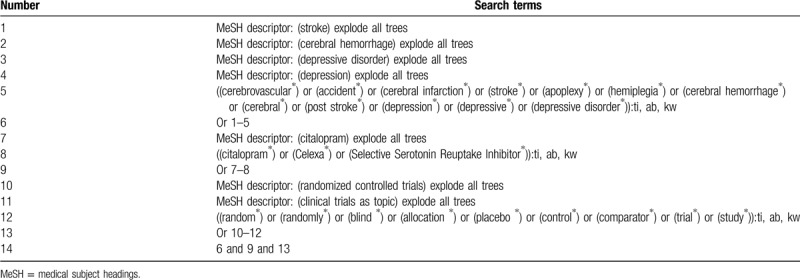
Search strategy of Cochrane library.

#### 2.3.2 Other literature resources

We will also identify any potential trials from other relevant literature resources, such as conference proceedings, and reference lists of included trials or connected reviews.

### 2.4 Study identification

All sought resources will be imported into EndNote 7.0 to remove any duplicates. Titles/ abstracts of remaining papers will be filtered by 2 independent investigators, and those clearly do not conform to the study eligibility criteria will be removed. After that, we will obtain full manuscripts of the potential trials and will carefully read whole documents. Any unqualified trials will be filtered out according to the full inclusion criteria. As for the literatures those can not be ensured, we will invite a third investigator by discussion and a final decision will be made after discussion. We will present the process of study identification in a flow diagram with details.

### 2.5 Data extraction and management

Two independent investigators will complete the data extraction from the qualified trials based on the data form uniformly developed by the experienced expert. Any uncertainty will be disentangled by the help of a third investigator through discussion or consultation. The extracted information is as follows:

General information: Title, first author, year of publication, contact information.

Participants: Age, ethnicity, sex, diagnostic criteria, inclusion and exclusion criteria, severity and duration of the disease.

Study methods: Study design, sample size, details of randomization, blind, and concealment.

Interventions and controls: types of modality, modality methods, dosage, period, and frequency.

Outcome indicators: primary and secondary outcomes, and number of adverse events.

Others: Funding sources, and conflict of interest.

### 2.6 Risk of bias assessment

As for study quality assessment, we will utilize Cochrane risk of bias tool, which is the recommended tool to appraise the risk of bias in RCTs in Cochrane Reviews. It covers different aspects of study design, conduct, and reporting through 7 domains. Each 1 is graded as low, unclear or high risk of bias. Two investigators will appraise them independently for each eligible trial, and any differences will be fixed with the help of a third investigator.

### 2.7 Statistical analysis

#### 2.7.1 Data synthesis

RevMan 5.3 software will be employed for statistical analysis. According to the different types of statistical data, binary categorical values will be expressed as risk ratio and 95% confidence intervals, and continuous values will be calculated as mean difference or standardized mean difference and 95% confidence intervals. *I*^*2*^ statistic test will be used to explore the heterogeneity of the research results. *I*^*2*^ ≤50% means low level of heterogeneity, and a fixed-effects model will be applied. If possible, we will conduct a meta-analysis if sufficient data are collected from the eligible trials with similar study characteristics, intervention and control modalities, and outcome indicators. Otherwise, *I*^*2*^ >50% suggests high level of heterogeneity, and a random-effects model will be exerted. Additionally, we will perform subgroup analysis to explore any possible causes for the high level of heterogeneity. If a meta-analysis can not be conducted, we will report findings using a descriptive summary.

#### 2.7.2 Subgroup analysis

Subgroup analysis will be performed based on the different types of general study information, patient characteristics, interventions, comparators, and outcome indicators.

#### 2.7.3 Sensitivity analysis

Sensitivity analysis will be carried out to check whether the findings are robust according to the sample size, study quality and statistical model.

#### 2.7.4 Reporting bias

If at least 10 included trials entered in a meta-analysis, we will investigate the potential reporting bias using funnel plot and Egger regression test.^[36,37]^

### 2.8 Quality of evidence

Quality of evidence for each outcome indicator will be appraised by Grading of Recommendations Assessment, Development, and Evaluation,^[38]^ and each level of evidence will be judged as very low, low, moderate, or high level.

### 2.9 Ethics and dissemination

This study will not need ethical approval, because we will only use published data. We will publish this study on a peer-reviewed journal.

## 3 Discussion

In recent years, the clinical RCTs of citalopram for the treatment of PSD have been increasing, but their conclusions are still contradictory at literature level.^[16–34]^ Thus, this systematic review will summarize the available direct high quality evidence to assess the efficacy and safety of citalopram for the treatment of PSD. Its results will provide literature evidence to judge whether citalopram is effective for the treatment of PSD, which may benefit clinical practice and health-related policy makers.

## Acknowledgment

This study was partly funded by Yan’an Science and Technology Research and Development Plan Project (2018KS-27). The funder had no role in this study.

## Author contributions

**Conceptualization:** Jing Hu, Zeng-ye Yang.

**Data curation:** Jing Hu, Zeng-ye Yang.

**Formal analysis:** Jing Hu, Li Ma, Zeng-ye Yang.

**Investigation:** Zeng-ye Yang.

**Methodology:** Jing Hu, Li Ma.

**Project administration:** Zeng-ye Yang.

**Resources:** Jing Hu, Li Ma.

**Software:** Jing Hu, Li Ma.

**Supervision:** Zeng-ye Yang.

**Validation:** Jing Hu, Li Ma, Zeng-ye Yang.

**Visualization:** Jing Hu, Li Ma, Zeng-ye Yang.

**Writing – original draft:** Jing Hu, Li Ma, Zeng-ye Yang.

**Writing – review & editing:** Jing Hu, Li Ma, Zeng-ye Yang.
